# An extensive arterial thrombosis with lower limb ischemia in a COVID‐19 patient: A case report

**DOI:** 10.1002/ccr3.8231

**Published:** 2023-11-20

**Authors:** Johary Andriamamonjisoa Andriamizanaka, Etienne Rakotomijoro, Volatiana Andriananja, Mihaja Raberahona, Radonirina Lazasoa Andrianasolo, Rivonirina Andry Rakotoarivelo, Jean de Dieu Randria Mamy

**Affiliations:** ^1^ Department of Infectious Diseases, Faculty of Medicine University of Antananarivo, Joseph Raseta Befelatanana University Hospital Antananarivo Antananarivo Madagascar; ^2^ Department of Endocrinology, Faculty of Medicine University of Antananarivo, Joseph Raseta Befelatanana University Hospital Antananarivo Antananarivo Madagascar; ^3^ Department of Infectious Diseases, Faculty of Medicine University of Fianarantsoa, University Hospital Tambohobe Fianarantsoa Fianarantsoa Madagascar

**Keywords:** arterial thrombosis, case report, COVID‐19, D‐dimer, ischemia

## Abstract

The coronavirus disease 2019 (COVID‐19) pandemic is responsible for huge morbidity and mortality throughout the world. Several serious complications of this disease have been reported. It can cause hypercoagulability, which may lead to venous and arterial thromboembolic diseases. This hypercoagulability state is also associated with high morbidity and mortality. Arterial thrombosis in COVID‐19 is poorly described compared to venous thrombosis and pulmonary embolism. We report a case of an extensive arterial thrombosis leading to a limb ischemia with extremely high D‐dimer in a COVID‐19 patient. A 69‐year‐old man was hospitalized for febrile dyspnea. He is a hypertensive and diabetic patient. On admission, pulse oxygen saturation was 72% on room air. He had cyanosis of the left foot up to the mid‐thigh. The left pedal, posterior tibial, popliteal and femoral pulses were abolished. Chest CT scan was in favor of COVID‐19. He has a high D‐dimer level of 257,344 ng/mL. Arterial Echo‐Doppler found an extensive intraluminal thrombus along the arterial axes of the left lower limb, completely obstructing them, starting from the primitive iliac artery just after its bifurcation with the aorta, and extending distally (external iliac; common femoral; superficial femoral; popliteal; anterior tibial; posterior tibial; fibular and pedal). The patient was diagnosed with COVID‐19 critical form, associated with ischemia of the left lower limb secondary to an extensive arterial thrombosis. He was receiving anticoagulation, and underwent surgical amputation of the ischemic limb. The patient survived the event; however, he was on long‐term oxygen therapy at home. Arterial thrombosis may occur during COVID‐19 and may be responsible for peripheral or central ischemia aggravating morbidity and mortality. The occurrence of these events is related to the D‐dimer value. Anticoagulation is an important part of the management of COVID‐19, especially in severe forms in order to limit the occurrence of these thromboembolic diseases.

## Graphical abstract

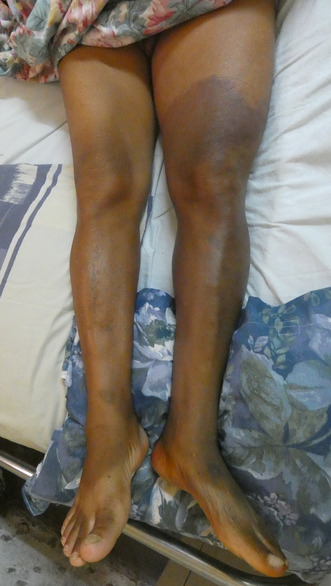

## 1 BACKGROUND

Coronavirus disease 2019 (COVID‐19) is a leading cause of morbidity and mortality worldwide. Apart from the respiratory complications which are responsible for most of the deaths, several serious complications have been reported including compressive emphysema or acute pancreatitis. COVID‐19 can also induce a hypercoagulable state, at the origin of venous and arterial thromboembolic events. This hypercoagulability is associated with high morbidity and mortality.^1^, ^2^ The literature mainly describes venous thromboembolic events and pulmonary embolisms.^3^, ^4^ Arterial thrombosis during COVID‐19 is poorly described compared to venous thrombosis. According to literature, it can occur with a prevalence of 0.13% in a hospitalized patient who tested positive for COVID‐19 and 0.19% in patients who tested negative.^5^ We report a case of an extensive arterial thrombosis with extremely high D‐dimer level that resulted in a lower limb ischemia in a COVID‐19 patient.

## 2 CASE PRESENTATION

**FIGURE 1:**
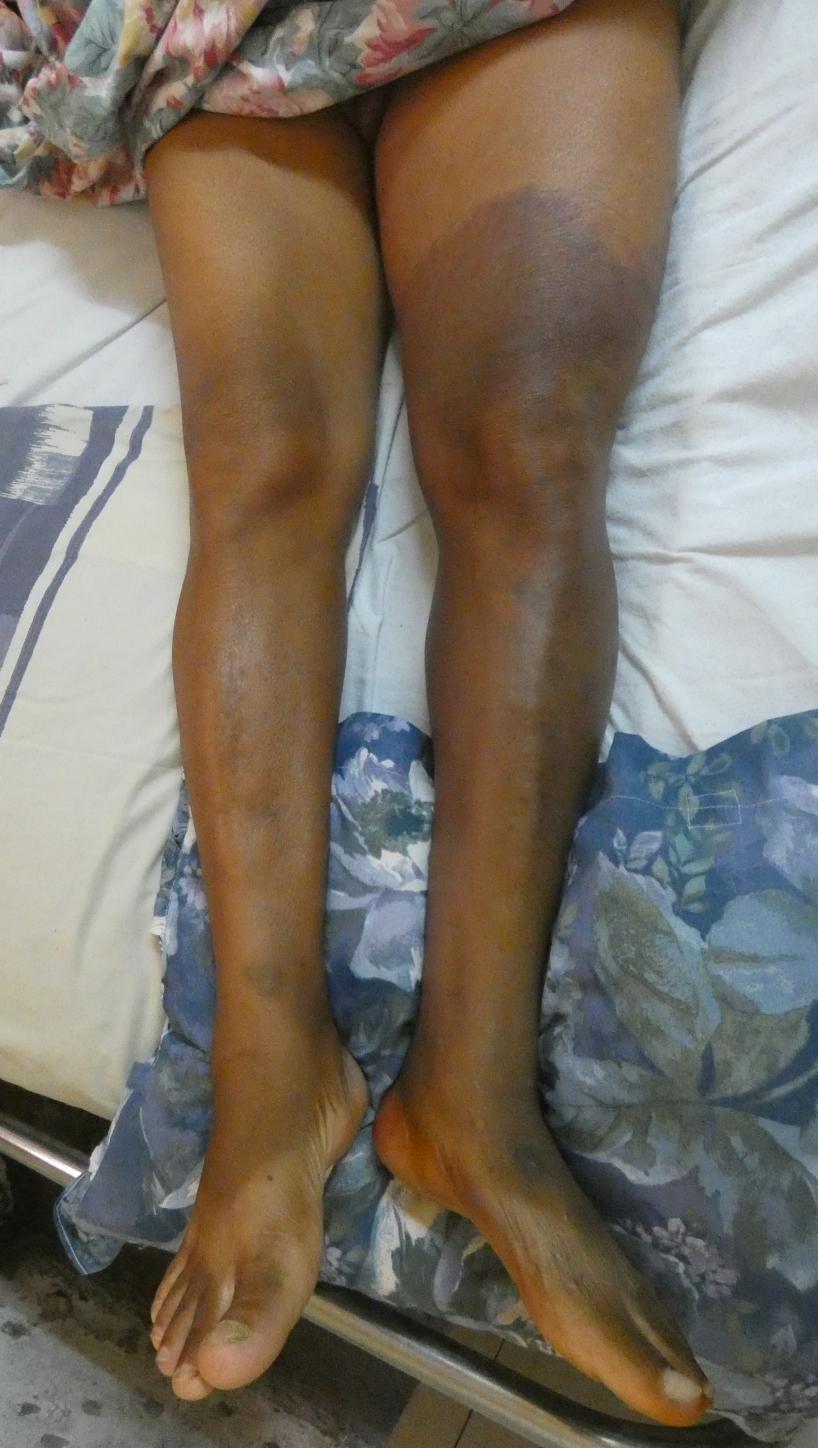
A photo of the patient's lower limbs shows the cyanosis from the left foot to the middle of the left thigh.

A 69‐year‐old man was hospitalized with febrile dyspnea at the military hospital of Antananarivo. He reported close contact with a confirmed case of COVID‐19 5 days before the onset of symptoms. His history included hypertension treated with Losartan 100 mg per day, type 2 diabetes treated with Metformin 1500 mg/day, alcoholism and smoking cessation for 10 years (9.5 pack‐years). He has no known history of peripheral artery disease. For 13 days prior to his admission, he presented with a dry cough, shortness of breath at rest without orthopnea, fever, asthenia, and anorexia. Two days before hospitalization, he felt a spontaneous severe pain with swelling of the left lower limb. Physical examination revealed a body mass index of 28.7 kg/m2, a pulsed oxygen saturation of 72% on room air, a respiratory rate of 23/min, a high temperature of 38.9°C, a heart rate of 117 beats/min, a blood pressure of 135/80 mmHg and bilateral clinical signs of pneumonia. He presented a cyanosis of the left foot up to the mid‐thigh (Figure 1), a hypoesthesia of the left lower limb and the skin was cool. The left pedal, posterior tibial, popliteal, and femoral pulses were abolished.

**FIGURE 2:**
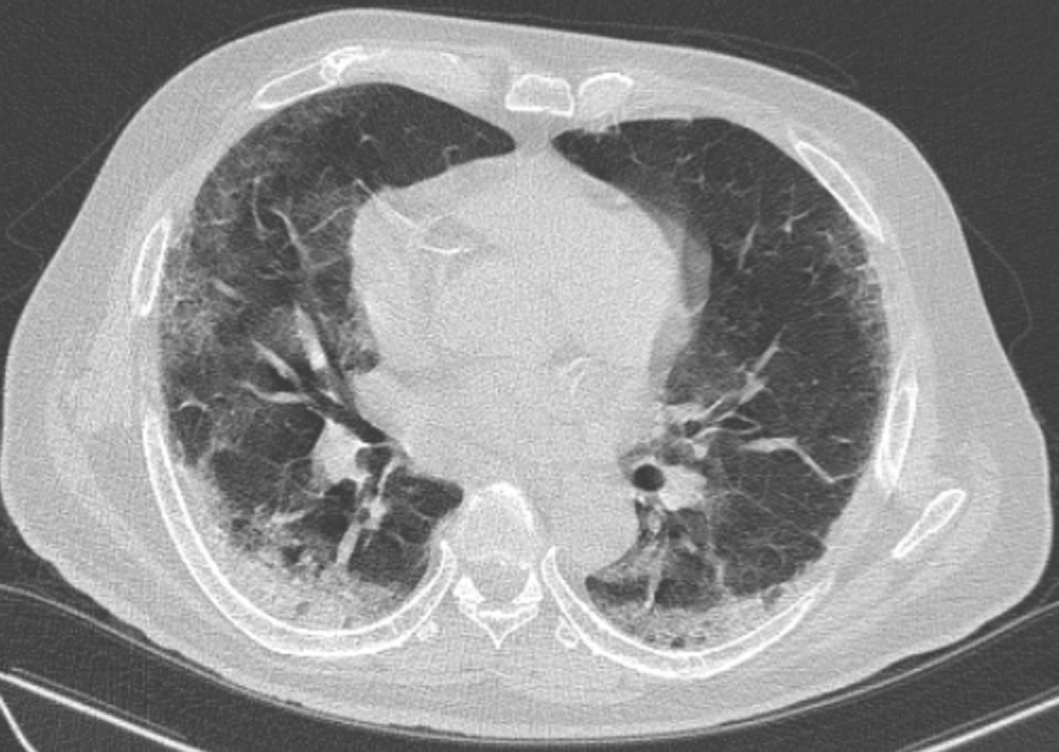
Chest CT scan without injection of contrast medium, axial section showing multiple extensive ground‐glass areas occupying 50–75% of the lung parenchyma, peripherally and centrally distributed, bilaterally subpleural, associated with fine intralobular reticulations giving a crazy paving appearance and areas of condensation, especially in the posterior basal segments of the lower lobe bilaterally.

**FIGURE 3:**
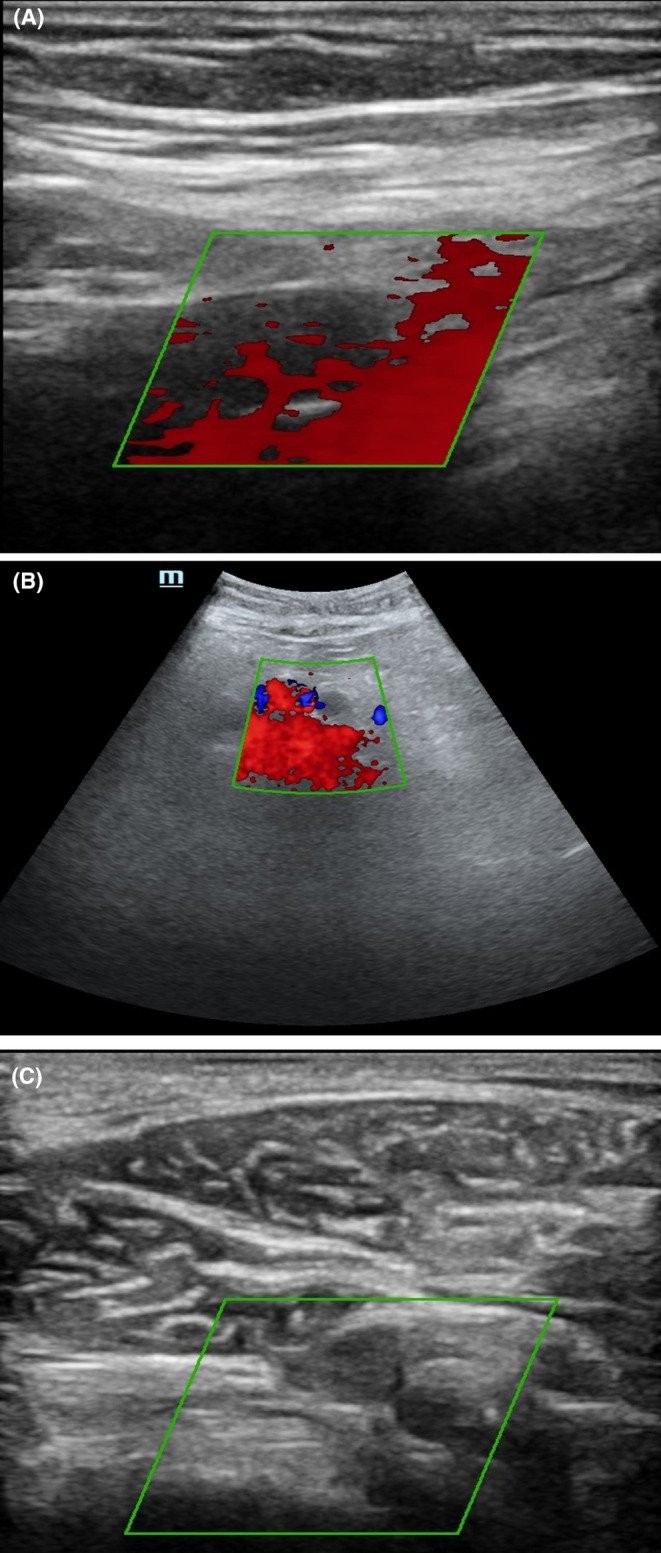
(A) Arterial Doppler ultrasound of the arterial axes of the left lower limb showing an extensive intraluminal thrombus along the arterial axes of the left lower limb, totally obstructive, starting from the primitive iliac artery just after its bifurcation with the aorta and extending distally (external iliac; common femoral; superficial femoral; popliteal; anterior tibial; posterior tibial; fibular and pedal). (B,C) Arterial Doppler ultrasound, other view of the left lower limb arterial thrombosis.

The nasopharyngeal swab for severe acute respiratory syndrome‐coronavirus‐2 (SARS‐CoV‐2) reverse transcriptase‐polymerase chain reaction (rt‐PCR) performed on admission was negative. The complete blood count showed a hemoglobin level of 13.3 g/dL (13.5–17.5 g/dL) a white blood cells count of 11.81 G/L (5–10 G/L) and a platelet count of 81 G/L (150–400 G/L). The C‐reactive protein was 82.6 mg/L (<6 mg/L). The creatinine was 187 μmol/L (65.4–119.3 μmol/L). The blood sodium level was 141 mmol/L (135–145 mmol/L) and the blood potassium level was 4.9 mmol/L (3.6–5.2 mmol/L). The glycated hemoglobin was 7.5% (<6%). The D‐dimer was 514 times the upper normal limit (220–500 ng/mL). The troponin was normal. The electrocardiogram showed a regular tachycardia with a heart rate of 103 bpm. Chest CT scan was in favor of COVID‐19 showing ground glass images with 50–75% involvement (Figure 2). The arterial doppler ultrasound showed an extensive intraluminal thrombus along the arterial axes of the left lower limb, completely obstructing them, starting from the common iliac artery just after its bifurcation with the aorta and extending distally (external iliac, common femoral, superficial femoral, popliteal, anterior tibial, posterior tibial, fibular, and pedal), without any detectable collateral circulation (Figure 3A–C). The patient was diagnosed with a severe COVID‐19 associated with acute ischemia of the whole left lower limb secondary to an extensive arterial thrombosis. He was receiving oxygen therapy with a high concentration oxygen mask at 15 L/min, corticosteroid therapy with intravenous dexamethasone (12 mg/day), subcutaneous therapeutic anticoagulation with enoxaparin at a curative dose (8000UI ×2/day), oral antibiotic therapy with levofloxacin (1 g/day) and insulin therapy (rapid‐acting insulin 14UI ×3/day and long‐acting insulin 20 UI/day). The patient was transferred to the surgical ward due to aggravation of the ischemia with skin necrosis of extremities and underwent an amputation of the ischemic left lower limb. The post‐operative follow‐up was simple. The patient was discharged after 28 days of hospitalization and was under long‐term oxygen therapy at home. At 1 month follow‐up, he remained well and there was no recurrence of other ischemia.

## 3 DISCUSSION AND CONCLUSION

We report here a case of a hypertensive and diabetic patient presenting a severe form of COVID 19 associated with an ischemia of the left lower limb with an extremely high D‐dimer level.

High D‐dimer value is associated with severe infection and high risk of thromboembolic events during COVID‐19.^6^ Arterial occlusion can be life threatening or can lead to significant disabilities. Our patient had a history of hypertension, previous smoking, and type 2 diabetes. According to the literature, these comorbidities are significantly associated with the occurrence of arterial thrombosis during COVD‐19.^5^ He was diagnosed with a severe form of COVID‐19 and presented at the same time an ischemia of the left lower limb secondary to an extensive thrombosis of several arteries in the left lower limb. Our patient had a D‐dimer up to 514 times the upper normal level. The D‐dimer level correlates with the severity of the disease and is a reliable prognostic marker of in‐hospital mortality in those admitted with COVID‐19.^6^, ^7^, ^8^ The risk of death is therefore high for our patient. In COVID‐19, arterial thrombosis mainly involves the heart, the brain, the kidney and the bowels. There were only few reported cases of arterial thrombosis involving limbs including the left arm, the left leg, the right arm, and the right leg.^5^ However, our case involved the entire left lower limb (thigh, leg, and foot).

Gold DD. et al. reported a series of cases of arterial thrombosis. The first case had a thrombosis of the two renal arteries, the superior mesenteric artery and the celiac trunk. The second case had a thrombosis of the aorta, the celiac trunk, hepatic, and the splenic arteries. The third case had a cerebral, pulmonary, splenic, and renal thrombosis. The fourth case had a thrombosis of the lung, the spleen, and the kidney. The last case was a peripheral thrombosis with an occlusion of the radial and ulnar arteries.^9^ All five patients had arterial occlusions that were life‐threatening or disabling, and four of the five patients did not survive. Despite the severity of the COVID‐19 and the arterial occlusion, our case had survived. However, the delay in the management led to a total obstruction of the artery causing ischemia which resulted in amputation. The outcome was unexpected given the severity of COVID‐19 and the extensive arterial occlusions in a country like Madagascar with limited technical platform and resources.

One limitation associated with this case report is that we have not been able to perform any serology test for covid 19 to strengthen the diagnosis made by chest CT scan, due to unavailability of this test. Farther hypercoagulable workup aside from D‐dimer should have been done also, but could not be due to the patient's financial situation and the country's technical facilities. Some tests were not available. As a result of this challenge, it was mainly the tests that were available and that the patient could afford that were carried out.

We report a case of an extensive peripheral arterial thrombus with an extremely high D‐dimer during COVID‐19. Arterial thrombosis can occur during COVID‐19, causing peripheral or central ischemia with increased morbidity and mortality. The occurrence of these events is related to the D‐dimer value.^10^ Thus, this report underlines the potential role of anticoagulation in COVID‐19 especially in severe forms and furthermore in patient with cardiovascular comorbidities, or in the case of a high D‐dimer value in order to limit the occurrence of thromboembolic diseases.

## AUTHOR CONTRIBUTIONS

**Johary Andriamamonjisoa Andriamizanaka:** Methodology; validation; visualization; writing – original draft; writing – review and editing. **Etienne Rakotomijoro:** Investigation; methodology; validation; writing – original draft; writing – review and editing. **Volatiana Andriananja:** Validation; visualization; writing – review and editing. **Mihaja Raberahona:** Validation; visualization; writing – review and editing. **Radonirina Lazasoa Andrianasolo:** Validation; visualization; writing – review and editing. **Rivonirina Andry Rakotoarivelo:** Validation; visualization; writing – review and editing. **Jean de Dieu Randria Mamy:** Validation; writing – review and editing.

## FUNDING INFORMATION

None.

## CONFLICT OF INTEREST STATEMENT

None of the authors declare any conflict of interest.

## CONSENT

Written informed consent was obtained from the patient to publish this report in accordance with the journal's patient consent policy.

## Data Availability

The photo, the anonymised CT and Doppler ultrasound sections are available from the corresponding author upon reasonable request.
